# *In silico* and pharmacokinetic assessment of echinocystic acid effectiveness in Alzheimer's disease like pathology

**DOI:** 10.2144/fsoa-2023-0150

**Published:** 2024-05-23

**Authors:** Ankul Singh S, Chitra Vellapandian

**Affiliations:** 1 Department of Pharmacology, SRM College of Pharmacy, SRM Institute of Science & Technology, Kattankulathur, Tamil nadu, 603203, India (Bharat)

**Keywords:** Alzheimer's disease, anti-inflammatory, echinocystic acid, *in silico*, lipid metabolism

## Abstract

**Aim:** Alzheimer's disease causes dementia which impairs the cognitive domains. **Methodology:** The pharmacokinetic characteristics and biological activity of echinocystic acid are predicted in this work using *in silico* or computational approaches, including pkCSM, Swiss ADME, OSIRIS^®^ property explorer, PASS online web resource and MOLINSPIRATION^®^ software. **Results & discussion:** The compound has lipid metabolism regulating property as major role in decreasing the progression of Alzheimer's disease and it has no major side effects and ADR. The drug also has anti-inflammatory properties which can help in regulating the innate immunity that plays a major role in Alzheimer's disease. **Conclusion:** From the computational screening, we infer that, echinocystic acid can regulate memory loss, cognitive disability and also slow down the progression of Alzheimer's disease-like pathology.

## Plain language summary

Alzheimer's disease (AD) is a neurodegenerative disease that is characterized by β-amyloid (Aβ) plaque deposition and neurofibrillary tangles of hyperphosphorylated tau. There is no treatment to completely cure AD and dementia but the progression of the disease can be slowed down and the major symptoms can be treated. Various online servers and web resources were employed in this study. The use of online and offline tools for the prediction and evaluation of the various drug properties and parameters have led to evidential conclusion of the study. The calculated binding affinities for all of the designed compounds range from -1.5 to -6.0 kcal/mol-1, while few receptors showed positive binding affinity indicating less binding with receptor. From the *in silico* study performed we infer that echinocystic acid can regulate memory loss, cognitive disability and slow down the progression of Alzheimer's disease like pathology.

## Abstract (plain language summary)

Bcl-2 protein, Cox-1 and IL-6 showed highest binding affinity in contrast to other target proteins.

Van der Waals and conventional hydrogen bond interactions were seen well in Bcl2, Cox-2 and AchE indicating its strong interaction with target proteins.

However, Bcl2, IL6, Cox1, NFκB, Cox2, Il-1β and AchE showed considerable bonding with receptors.

Further, *in vitro* assays and *in vivo* techniques can be studied in depth to understand the potential of this compound in neurodegenerative like diseases.

Neurodegenerative diseases are characterized by the gradual loss of selectively sensitive groups of neurons in contrary to selective static neuronal loss caused by metabolic or toxic illnesses [1]. Alzheimer's disease (AD) is a neurodegenerative disease that is characterized by β-amyloid (Aβ) plaque deposition and neurofibrillary tangles of hyperphosphorylated tau. The disease causes dementia which impairs the cognitive domains. The cognitive domain includes memory, language, behavioral and personality changes and visuospatial function [2]. The diagnosis Of AD is done by laboratory imaging techniques like CT, PET, MRI and biomarkers including beta-amyloid 42, tau and phosphorylated-tau besides physical and neuronal examinations [3]. Neuropsychological and mental tests are also performed in order to check the memory and thinking ability. While performing the physical examinations, muscle tone, reflexes, sense to sight and hearing, co-ordination, and balance are monitored [4]. The plaques and tangles in the brain are imaged using magnetic resonance imaging, CT scan. The above-mentioned test is used to study the neuronal shrinkage and degeneration in the brain. The patterns of degeneration, amyloid deposition and neurofibrillary tangles are traced using positron emission tomography.

The disease progresses with specific neuropathological findings like synapse loss, specific neuron loss, neurophil threads. The major indications are senile plaques and neurofibrillary tangles which look like plaques [5]. The plaques are found in between the dying brain cells and the tangles are found on the nerve tissues as tiny spots. The β-amyloid peptide deposition is due to the mutation in the precursor proteins of β-amyloid peptide or in presenilin-1 (PS1) or presenilin-2 (PS2). This is believed to be the reason for early onset of Alzheimer's disease where the ratio of Aβ is increased. The Aβ formation and deposition is enhanced by the hike in free cholesterol level in the neuronal cell membrane [6]. Factors like aging, metabolic disorders or co-morbid conditions and excitotoxicity may result in imbalance in the formation and clearance of Aβ [7]. The clearance of Aβ is mediated by low density lipoprotein receptor related-1 and some of the studies show that p-glycoprotein also plays a major role in clearance of the Aβ at bloodߝbrain barrier efflux pump [8,9].

There is no treatment to completely cure AD and dementia but the progression of the disease can be slowed down and the major symptoms can be treated [10]. The cognitive symptoms can be treated using FDA approved drugs: donepezil, galantamine, rivastigmine. Glutamate regulators; memantine helps in regulating glutamate which improves neural communication [11]. These drugs are cholinesterase inhibitors and they help in increasing the neural communication. Treating the emotion and behavioral changes in Alzheimer's disease is also important. Anti-depressants, anti-psychotics, and anti-anxiety drugs are given to cope with mood swings, delusions, aggression, and hallucination. Aducanumab is an anti-amyloid antibody administered intravenously to prevent the deposition of Aβ [12].

*Luffa cylindrica* belonging to Cucurbitaceae family has its synonyms like *Luffa insularum*, *Luffa petola*, *Luffa subangulata*, *Luffa sylvestris*, *Momordica cylindrica*, *Momordica Luffa*, *Momordica Luffa* etc. They are mostly found in Africa, Asia (tropic and sub-tropic areas). Leaves, flowers, and fruits of the plant are used medicinally. *Luffa cylindrica* screening for phytochemical identification indicate that it includes anthocyanins, glycosides, flavonoids, triterpenoids, cardiac glycosides, saponins, carbohydrates, proteins, alkaloids, and tannins. Echinocystic acid, a triterpenoid sapogenin derived from *Luffa cylindrica*, had immunomodulatory properties [^13-15^].

Using computational techniques, this work examines the neuroprotective and anti-inflammatory properties of echinocystic acid, a triterpenoid sapogenin found in Luffa cylindrica. Analyses and summaries of the pharmacokinetic parameters, probable negative effects, and toxicity tests were performed. In this investigation, a variety of online servers and web resources were used. The study's conclusive evidence came from the use of online and offline technologies for the evaluation and prediction of the numerous pharmacological features and parameters. Further research on this subject may benefit greatly from the evidential evidence as well. The use of *in silico* methods for analyzing the drug candidate's pharmacology provides a clear grasp of the properties of absorption, distribution, metabolism, excretion and toxicity. As a result, innovative compounds with affinity for the target region can be improved.

## Methodology

Different *in silico* tools and software can be used to study the physicochemical properties, pharmacokinetic properties, and pharmacological activity of EA and its significance in becoming a part of treatment in AD-like pathology. The tools used for the assessment of EA and their significance is discussed below. *In silico* molecular docking also plays an essential role in predicting the affinity of the drug with the macromolecule. Thus, *in silico* portion have been discussed in context with the studied activity in pharmacokinetic evaluation by using online tools. This study will signify the need to carry out computational techniques and understand the biology of drug and further obtaining it as supportive evidence to carry on further research work both *in vitro* and *in vivo* studies, respectively.

### *In silico* molecular docking

The crystal structures were retrieved from the Protein Data Bank (http://www.rcsb.org/). As a receptor molecule, various targets including Cox 1 (PDB 6Y3C), Cox 2 (PDB 5F19), NFκB (PDB 6M2D), IL-1 beta (PDB 5R8Q), IL 6 (PDB 1ALU), Bcl-2 (PDB 5UUK), Caspase 3 (PDB 4JJE), AchE (PDB 2GYU), BDNF (PDB 1B8M) and CREB (PDB 5I86) was selected. The inhibitor ligand and the remaining other chains were removed from the PDB structure. The proteins for this investigation were created using the MMV and Autodock tools. After the water molecules were removed and hydrogen atoms were supplied to stabilize the tautomeric and ionised states of the amino acid residues, the upgraded protein was further improved. The corrected protein structure was then saved as a pdb file and used in all subsequent docking experiments. The Molegro molecular viewer was used to optimize the proteins used in this investigation. After water and ligands that were covalently connected were removed, the bond order was altered. Utilizing the molecular mechanics force field, energy reduction was accomplished after assigning charge and protonation states. The AutoDock application automatically computes Gasteiger charges and analyses the rotatable bonds of the ligand to create several conformers for docking. The grid points of each designated receptor were used to build the receptor grids, and the grid boxes were generated utilizing those axes as a domain. The macromolecule was in the main core of the grid box. Autogrid 4 was used to generate the map types. For all molecular docking simulations, the Lamarckian genetic technique was utilized [^16-19^]. Docking was performed with the following parameters: 50 runs, 150 population members, 2,500,000 evaluations and 27,000 generations. The docking snap images were imported using the Biovia discovery studio 2019, and the final docked structures were exported in pdb format. Hydrogen and hydrophobic interactions at the inhibitor sites of docked targets were observed to view the docking findings using Biovia Discovery Studio 2019.

### Estimation of pharmacokinetic profile

**Figure 1.**
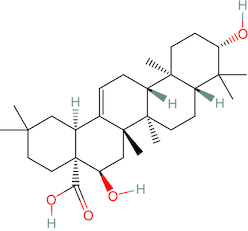
Docked compound echinocystic acid with various targets related to Alzheimer's disease like pathology.

Using data from the PubChem web server, the canonical smiles of echinocystic acid are shown in Figure 1. Utilizing pkCSM and Swiss ADME, the pharmacokinetic investigations were completed [^20-22^]. Using the canonical smiles, the ADMET profile is extracted from the web server. The drug's absorption, distribution, metabolism, excretion, and toxicity profile are provided by the pkCSM and Swiss ADME. The online tool pkCSM is effective at studying the pharmacokinetic characteristics of medications. Utilizing the pkCSM tool, N-benzoyl thiourea derivatives were assessed for their physicochemical characteristics and analgesic efficacy [23].

### *In silico* prediction of toxicity

Toxicological testing is crucial in the drug withdrawal process in order to avoid consequences like organ system failure or damage. The chemical structure of the compounds was referenced to from PubChem in order to determine the toxicity profile using the OSIRIS^®^ property explorer programme. A color scale is used to assess the tumorigenicity, mutagenicity, irritant, reproductive effect and drug-likeness of substances [24] TPSA, drug-likeness, overall drug score was generated.

- Green: high risk of toxicity;
- Yellow: medium risk of toxicity;
- Red: high risk of toxicity.

### Prediction of biological activity of echinocystic acid using PASS online server

The biological activity of the medications was predicted using the online tool Pass (Prediction of Activity Spectra for Substances). Based on the medications' structural formula, the biological activity spectrum of the drugs is projected. The analysis is solely based on the link between structure and activity [25]. Pharmacological/biological activity plays a significant part among the qualities of chemical compounds since it offers usage for the compounds in medical applications [26]. Using the PASS ONLINE website resource, the side effects of the medications were also anticipated. In order to evaluate the biologically active substances, PASS online software is employed. More than 300 pharmacological actions and biochemical mechanisms can be predicted by the software. The forecast is based on the natural substance's structural formula. This could assist us in identifying novel pharmacological targets [27].

### Molecular property assessment

Utilizing MOLINSPIRATION^®^ software, *in silico* testing of the chosen medications was done to assess drug similarity and forecast bioactivity [28]. The bioactivity score ranges from -0.50 to 0.00 shows moderate activity and 0.00 suggests the medicine is inactive. Drugs with a bioactivity score of <0.00 are said to have optimum biological activity.

## Results & discussion

### *In silico* molecular docking

A molecular docking simulation investigation of the intended inhibitors was carried out utilising echinocystic acid on the receptor (PDB code: 5UUK) and other targets. All of the proposed compounds have computed binding affinities that vary from -1.5 to -6.0 kcal/mol-1, although few receptors displayed positive binding affinities, indicating less interaction with receptor. This indicates that only a small number of the selected receptors showed exceptional biochemical interactions at the binding region of the protein target. Using the Discovery Studio Visualizer programme, several of the high binding energy targets were examined to determine the kinds of molecular interactions and amino acid residues in charge of the biochemical interactions noticed at the receptor's binding site. Table 1 & Figure 2 shows the results of molecular docking simulations for certain targeted receptors and in most of the chosen targets, shorter hydrogen bond lengths (less than 3.0) were linked with a considerable increase in the number of amino acid residues involved in hydrogen bonding and van der waals interactions.

Figure 2.Docked compound echinocystic acid with various targets related to Alzheimer's disease like pathology.

**Figure:**
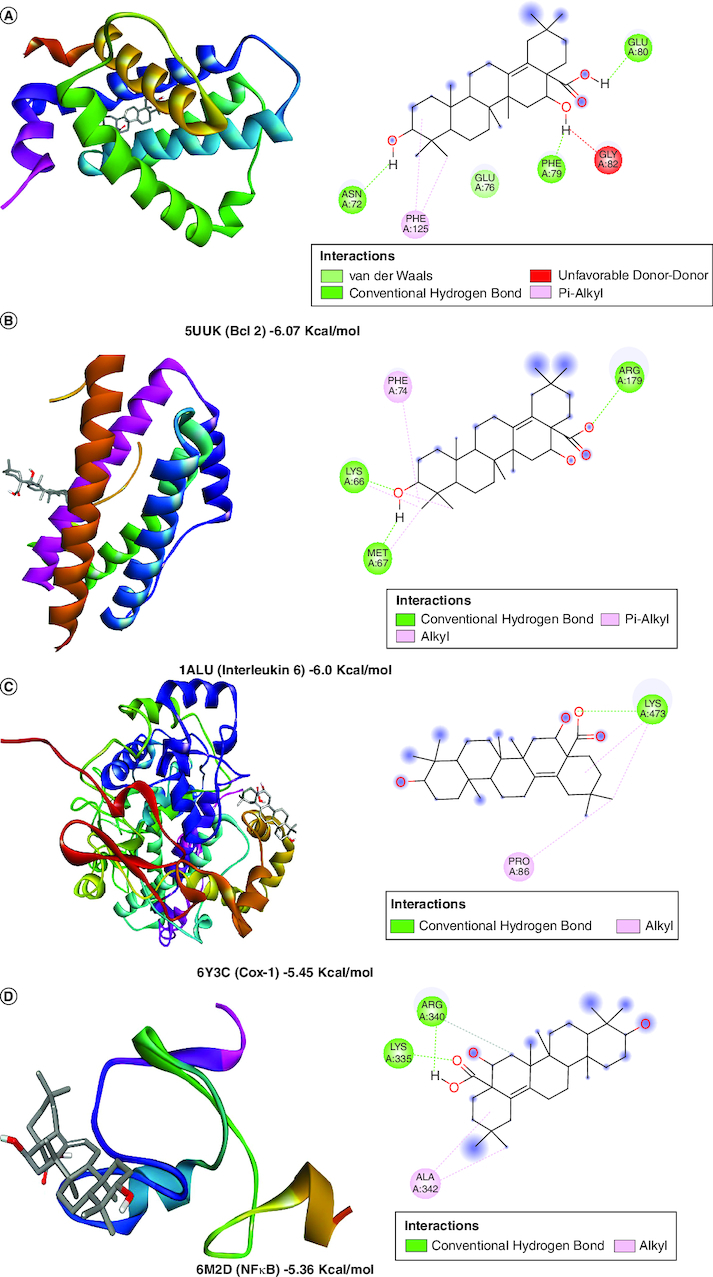

**Figure:**
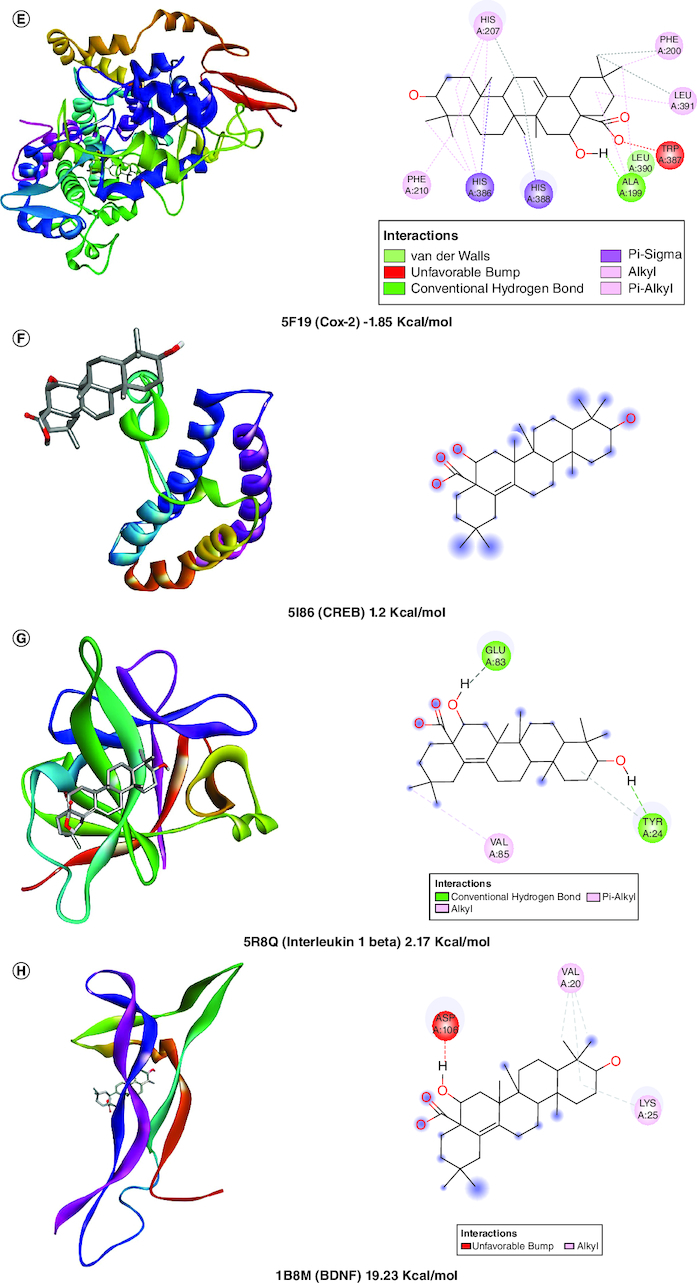

**Figure:**
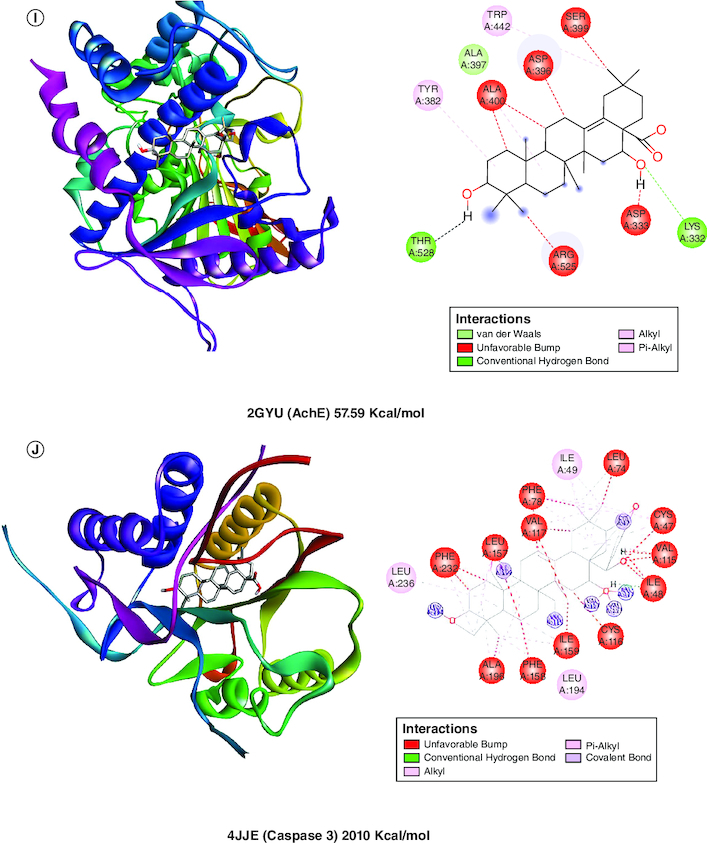

**Table 1.** Evaluation of *in silico* activity of echinocystic acid on Alzheimer's disease-like pathology.

Protein name	PDB ID	Binding energy (Kcal/mol)	Amino acid residues	Interaction
Bcl 2	5UUK	-6.07	GLUA76, GLUA80, PHEA79, ASNA72	Van der Waals, conventional hydrogen bond
IL6	1ALU	-6.0	ARGA179, LYSA66, META67	Conventional hydrogen bond
Cox 1	6Y3C	-5.45	LYSA473	Conventional hydrogen bond
NFκB	6M2D	-5.36	ARGA340, LYSA335	Conventional hydrogen bond
Cox 2	5F19	-1.85	LEUA390, ALA199	Van der Waals, conventional hydrogen bond
CREB	5I86	1.2	–	Unfavorable hydrogen bond
Il-1β	5R8Q	2.17	GLUA83, TYRA24	Conventional hydrogen bond
BDNF	1B8M	19.23	–	Unfavourable hydrogen bond
AchE	2GYU	57.59	THRA528, LYSA332	Van der Waals, conventional hydrogen bond
Caspase 3	4JJE	2010	–	Unfavourable hydrogen bond

### Estimation of pharmacokinetic profile

The developed inhibitors' pharmacokinetic characteristics and drug-likeness predictions based on their binding affinities demonstrated that the best-docked compounds are considered viable drug candidates. However, while employing proper methodologies in drug design, development and discovery expeditions, it is necessary to include some crucial pharmacokinetic characteristics or ADMET attributes (absorption, distribution, metabolism, excrétion and toxicity) as the most crucial characteristics [29]. As a result, the physicochemical parameters of the evaluated compound (Table 2) and ADMET features of the evaluated compound (Tables 3, 4 & 5) were thoroughly investigated using online web-based tools. For the analysed compound, the predicted physicochemical features that have a substantial impact on a molecule's effectiveness, safety, or metabolism were analysed using Lipinski's rule of five. The findings showed that Consensus Log Po/w, Log Po/w (WLOGP), and Log Po/w (XLOGP3) all had log P violations. XLOGP3, WLOGP and MLOGP, which are atomistic and topological implementations of Moriguchi's topological method, respectively, were used to observe the logarithm of the partition coefficient between n-octanol and water for lipophilicity between -0.7 and +5.0. A consensus log Po/w value is the arithmetic mean of the five above-mentioned predictive values, and SILICOS-IT is the log Po/w estimation by hybrid approach of fragments and topological descriptors [30]. This shows that all of the suggested inhibitors have pharmacological or drug-like properties that enable oral administration. The projected ADMET properties are provided clearly in Table 3. Echinocystic acid has a negligible solubility in aqueous solutions, which ultimately impairs the compound's distribution and absorption. The high intestine absorption of 94.9%, however, is above the average range of intestinal absorption. EA was discovered to have a bloodߝbrain barrier permeability of -0.544 (log BB). Its CNS permeability, however, was discovered to be -1.448 (log PS), suggesting that it has improved CNS penetration and can exercise its pharmacological effects. The computed absorption properties (percent human intestine absorption, skin permeability, and BBB permeability), which were based on ADMET predictions, were discovered to be within the threshold values and to not be P-glycoprotein II inhibitors. This shows that EA were well absorbed by humans and have important pharmacological characteristics. Because their predicted values were within the limit, EA had a desirable physiochemical profile.

**Table 2.** Pharmacokinetic profile of echinocystic acid.

Parameter	Predicted value
Water solubility (log mol/l)	-3.109
Intestinal absorption (human) (% absorbed)	94.939
P-glycoprotein I/II inhibitor	No
VDss (human) (log l/kg)	-1.217
Fraction unbound (human) (Fu)	0.065
BBB permeability (log BB)	-0.544
CNS permeability (log PS)	-1.448
CYP2D6 substrate	No
CYP3A4 substrate	Yes
CYP1A2/2C19/2C9/2D6/3A4 inhibitor	No
Total clearance (log ml/min/kg)	-0.043
Maximum tolerated dose (human) (log mg/kg/day)	0.034
hERG I/II inhibitor	No
Oral rat acute toxicity (LD_50_) (mol/kg)	2.227
Oral rat chronic toxicity (LOAEL) (log mg/kg/day)	1.808

**Table 3.** Physicochemical properties of echinocystic acid using swiss ADME.

Physicochemical Properties	
Parameter	Value
Molecular weight	472.70 g/mol
Heavy atoms (n)	34
Arom. heavy atoms (n)	0
Fraction Csp3	0.90
Rotatable bonds (n)	1
H-bond acceptors (n)	4
H-bond donors (n)	3
Molar refractivity	137.82
TPSA	77.76 Å^2^

**Table 4.** Lipophilicity profile of echinocystic acid profile using swiss ADME.

Lipophilicity	
Parameter	Value
Log *P*_o/w_ (iLOGP)	3.47
Log *P*_o/w_ (XLOGP3)	6.88
Log *P*_o/w_ (WLOGP)	6.20
Log *P*_o/w_ (MLOGP)	4.97
Log *P*_o/w_ (SILICOS-IT)	4.96
Consensus Log *P*_o/w_	5.30

**Table 5.** Water solubility profile of echinocystic acid using swiss ADME.

Water solubility	
Parameter	Value
Log S (ESOL)	-7.04
Solubility	4.32e-05 mg/ml; 9.14e-08 mol/l
Class	Poorly soluble
Log S (Ali)	-8.32
Solubility	2.24e-06 mg/ml; 4.75e-09 mol/l
Class	Poorly soluble
Log S (SILICOS-IT)	-5.30
Solubility	2.37e-03 mg/ml; 5.02e-06 mol/l
Class	Moderately soluble

The overall clearance of echinocystic acid, a CYP3A4 substrate, was found to be -0.043 ml/min/kg. 0.034 mg/kg/day of echinocystic acid is the highest dose that can be tolerated by humans, according to data from the pkCSM web server. Echinocystic acid's LD_50_ was determined to be 2.227 mol/kg. Skin sensitization or hepatotoxicity are not present in the chemical. Utilizing Molinspiration, the bioactivity score of a few novel substituted imidazoline compounds was investigated. The study demonstrates that this pharmacokinetic method is reliable for performing computational drug development and pharmacokinetic analysis because it produced a favourable bioactivity score for therapeutic targets [31]. Echinocystic acid has a high GI absorption, according to the pharmacokinetic profile of the substance determined by Swiss ADME. It is a substrate for p-glycoprotein. The pkCSM server generated all of the ADMET parameters [32].

### *In silico* prediction of toxicity

Utilising *in silico*, the toxicity of experimental inhibitors of the studied inhibitors was determined. Using OSIRIS Property Explorer, the compound's physiochemical characteristics were identified. Before a drug candidate enters clinical trials, it is essential to do research and determine whether the toxicity profile is unbearable. The toxicity parameters of these studied compound (Table 6) revealed that the compound Echinocystic acid was safe and considered non-mutagenic, non-tumorigenic, Irritant free, and showed non-reproductive effects, respectively. The majority of the proposed inhibitors were shown to be safe as a significant treatment option as a result of the toxicity profiles. All of the aforementioned factors were given a green indication by the OSIRIS property explorer, enhancing its safety profile. The absorption of a medication is ultimately determined by the topological polar surface area (TPSA) of a molecule. Additionally, it aids in the drug's penetration and delivery [33]. The TPSA of the compound was observed to be 77.76 from the OSIRIS property explorer.

**Table 6.** Toxicity profile of echinocystic acid using OSIRIS.

Parameters	Echinocystic acid (scores)
Mutagenic	GREEN
Tumorigenic	GREEN
Irritant	GREEN
Reproductive effect	GREEN
TPSA	77.76
Drug likeness	-1.63
Drug score	0.33

The compound's reported drug likeness was -1.63. Positive results imply that the molecule comprises fragments that are primarily found in marketed medications on the market. The drug score is the result of adding a compound's molecular weight, toxicity hazards, and cLogP. The drug score is crucial because it helps determine whether a chemical component can fulfil the requirements of a potential medicine. Echinocystic acid was discovered to be 0.33 of the medication.

### Pass (prediction of activity spectra for substances) online web resource

Additionally, the compounds were examined using web-based resources for bioactivity and medicinal chemistry. Table 7 displays the predicted bioactivity scores as well as the medicinal chemistry characteristics of numerous developed drugs. It displayed a number of different behaviours, including anti-inflammatory, lipid metabolism regulator, lipid peroxidase inhibitor, hypolipemic, and acylcarnitine hydrolase inhibitor. All of these substances have already had their ligand-binding affinities evaluated (EA). Pa and Pi values indicate the likelihood of an event. Pa stands for the likelihood of being active, and Pi for the likelihood of being inactive. You can anticipate that the molecule will exhibit that action if Pa >0.71.

**Table 7.** Evaluation of biological activity of echinocystic acid using PASS online.

Pa (active probability)	Pi (inactive probability)	Biological activity
0.827	0.005	Anti-inflammatory
0.786	0.006	Lipid metabolism regulator
0.779	0.004	Lipid peroxidase inhibitor
0.770	0.009	Hypolipemic
0.710	0.025	Acylcarnitine hydrolase inhibitor

### Molecular property assessment

The *in silico* screening of the selected drugs were performed using MOLINSPIRATION^®^ software. Table 8 showed the bioactivity score of echinocystic acid. The bioactivity score of the drug was estimated for drug binding to nuclear receptor ligand, protease inhibitor, enzyme inhibitor, G-protein coupled receptor, ligand kinase inhibitor and ion channel modulator using Molinspiration. For the selected drugs, bioactivity ratings were assigned for the G protein-coupled receptor (GPCR) ligand, ion channel modulator, nuclear receptor ligand, kinase inhibitor, and enzyme inhibitor. As demonstrated in Table 8, all of the substances tested, including the referred drug, are active G protein-coupled receptor (GPCR) ligands with projected bioactive values greater than 0.00. PAINS alerts (pan assay interference) and synthetic accessibility for medicinal chemistry aspects were also investigated. None of the developed compounds had an alarm (PAINS alert = 0). A chemical is thought to have considerable biological activities if its bioactivity score is larger than 0.00. Bioactivity scores between -0.50 and 0.00 are thought to be moderately active, and bioactivity scores below -0.50 are thought to be inactive [31,34].

**Table 8.** Bioactivity score of echinocystic acid using molinspiration.

Parameter	Value
GPCR ligand	0.23
Ion channel modulator	-0.12
Kinase inhibitor	-0.41
Nuclear receptor Ligand	0.72
Protease inhibitor	0.17
Enzyme inhibitor	0.58

## Discussion

*In silico* computational technique are thus utilized as an alternative to animal testing to understand clearly about the potential response of an organism to a chemical stressor [35]. Various multi-target therapeutic approaches have been aimed at simultaneous targeting of multiple proteins involved in the development of a disease is recommended to get clear insight of disease [36,37]. Studies suggest that COX-1 and COX-2 are involved in the pathology of AD, a neuro-inflammatory dementia [38]. Intrinsic myeloid cells in the brain are involved in the neuro-inflammation process. Inflammation stands to be major factor in the early onset of AD. Studies suggest that treatments that are specific to immunity (innate immune system) and inflammation may protect the disease progression and inflammation driven onset of the neurodegenerative disease. The COX-2 pathway has its effect on the pathophysiology of AD and neurodegeneration [^39-42^]. From analyzing the activity of the compound echinocystic acid, it is revealed to possess anti-inflammatory effect which may help in slowing the disease progression and onset.

Dysfunction in lipid metabolism is the major factor in the pathology of AD. The imbalance in lipid composition may result in disruption of blood–brain barrier, altered myelination, defects in cell signalling, abnormality in the processing of amyloid peptide protein, disturbed energy metabolism, and neuro-inflammatory process in AD. High cholesterol diet leads to dyslipidemia which is a risk factor for AD. Mutation in apolipoprotein E gene may lead to defect in apolipoprotein E which in turn affects the clearance of the amyloid beta peptide in the brain. This may lead to initiation of pathogenesis of AD. Echinocystic acid possesses a property of regulating lipid metabolism and cholesterol composition [^43-46^]. The dose dependent anti-inflammatory, antiviral, and antioxidant activity of EA was evaluated in 2019 by Hailong Yu *et al.* The study demonstrated neuroprotective activity of EA in a mouse with cerebral ischaemia. Anti-apoptotic effect was exhibited by EA with anti-inflammatory effect by inhibiting the proliferation of NF-κB. EA also prevented elevation of IL-1β, IL-6 and signalling of JNK pathway. It was concluded that EA can be used in the treatment of cerebral ischaemia and brain injury in neurological disorders [47]. Many studies conducted on effect of lipid peroxidase causes cell damage and neuronal damage in the brain. The enzyme lipid peroxidase induces cell death in elderly AD patients. Free radical-mediated injury results in damage of cell membrane and production of secondary products. This processed by fission and endocyclization of oxygenated free fatty acids. These secondary products produce neurotoxicity [48].

Echinocystic acid inhibits this lipid peroxidase which may prevent the induction of cell death. In the year 2019, a study conducted by Hailong Yu *et al.* demonstrated anti-apoptotic effect due to up regulation of Bcl-2 protein and down regulation of cleaved caspase-3. The inhibition of JNK signalling pathway also had an effect on decreasing cell apoptosis and degeneration [49]. Loss of memory and cognitive disability can be regulated by acylcarnitine in case of AD. Acycarnitine also brings down the rate of progression of AD. Echinocystic acid acts as an acylcarnitine hydrolase inhibitor which prevents the degradation or breaking acylcarnitine which plays a major role in regulation of lipid metabolism, cell stress response [50,51]. A study conducted by Jung *et al.*, suggested that lancesamide A and its metabolite EA has a potential to the activity of acetylcholine esterase which is equal to the activity of donepezil. The memory loss and learning deficit was induced by scopolamine. When lancesamide is converted into its metabolite EA, it elevates brain derived neurotrophic factor and phosphorylated cAMP response element binding (CREB) protein which plays a major role in positive regulation of memory and thinking [52].

## Conclusion

Alzheimer's diseases characterized by beta-amyloid plaque deposition and neurofibrillary tangles can be delayed using some of the FDA-approved drugs. From the *in silico* study performed we infer that echinocystic acid can regulate memory loss, cognitive disability and slow down the progression of Alzheimer's disease like pathology with hydrogen bonding accounting for most interactions. It is helpful in preventing cell death caused by lipid peroxidase enzyme. In some *in vivo* models, echinocystic acid can be explored in detail for the treatment of Alzheimer's disease-like pathology due to its neuroprotective effects. Certain developed inhibitors' ADMET characteristics show that they have good absorption, distribution, metabolism and excretion characteristics, and none of the chosen inhibitors are toxic or carcinogenic. These findings could be used to develop and improve the proposed inhibitors as potential therapeutic medicines for the treatment of AD-like disease. For a deeper understanding of the processes behind their actions and other pharmacological effects, more experimental study and clinical trials are warranted.

## References

[1] Dugger BN, Dickson DW. Pathology of neurodegenerative diseases. Cold Spring Harb. Perspect. Biol. 9(7), a028035 (2017). 28062563 10.1101/cshperspect.a028035 PMC5495060

[2] Weller J, Budson A. Current understanding of Alzheimer's disease diagnosis and treatment. F1000Research 7, 1161 (2018). 10.12688/f1000research.14506.1 PMC6073093 30135715

[3] Blennow K, Zetterberg H. Biomarkers for Alzheimer's disease: current status and prospects for the future. J. Intern. Med. 284(6), 643–663 (2018). 30051512 10.1111/joim.12816

[4] Ouellette J, Lacoste B. From neurodevelopmental to neurodegenerative disorders: the vascular continuum. Front. Aging Neurosci. 13, 749026 (2021). 34744690 10.3389/fnagi.2021.749026 PMC8570842

[5] Murphy MP, LeVine H. Alzheimer's disease and the amyloid-β peptide. J. Alzheimer's Dis. 19(1), 311–323 (2010). 20061647 10.3233/JAD-2010-1221 PMC2813509

[6] Cheignon C, Tomas M, Bonnefont-Rousselot D, Faller P, Hureau C, Collin F. Oxidative stress and the amyloid beta peptide in Alzheimer's disease. Redox Biol. 14, 450–464 (2018). 29080524 10.1016/j.redox.2017.10.014 PMC5680523

[7] Suresh S, Begum RF, Singh SA, Chitra V. Anthocyanin as a therapeutic in Alzheimer's disease: a systematic review of preclinical evidences. Ageing Res. Rev. 76, 101595 (2022). 35217244 10.1016/j.arr.2022.101595

[8] Sadigh-Eteghad S, Sabermarouf B, Majdi A, Talebi M, Farhoudi M, Mahmoudi J. Amyloid-Beta: a crucial factor in Alzheimer's disease. Med. Princ. Pract. 24(1), 1–10 (2015). 10.1159/000369101 PMC5588216 25471398

[9] Singh AS, Vellapandian C. Structure of the bloodߝbrain barrier and its role in the transporters for the movement of substrates across the barriers. Curr. Drug Metab. 24(4), 250–269 (2023). 37291784 10.2174/1389200224666230608110349

[10] Singh AS, Vellapandian C. The role of plant-based products in the prevention of neurological complications. Drug Metab. Bioanal. Lett. 15(2), 81–92 (2022). 10.2174/1872312815666220413095159 35422230

[11] Howland RH. Drug therapies for cognitive impairment and dementia. J. Psychosoc. Nurs. Ment. Health Serv. 48(4), 11–14 (2010). 10.3928/02793695-20100311-01 20349884

[12] Bomasang-Layno E, Bronsther R. Diagnosis and treatment of Alzheimer's disease: Delaware. J. Public Heal. 7(4), 74–85 (2021). 10.32481/djph.2021.09.009 PMC8482985 34604768

[13] Singh AS, Vellapandian C. Phytochemical studies, antioxidant potential, and identification of bioactive compounds using GC-MS of the ethanolic extract of *Luffa cylindrica* (L.) fruit. Appl. Biochem. Biotechnol. 194(9), 4018–4032 (2022). 35583705 10.1007/s12010-022-03961-1

[14] Akinyinka Akinwumi K, Olusoji Eleyowo O, Omowunmi Oladipo O. A review on the ethnobotanical uses, phytochemistry and pharmacology effect of *Luffa cylindrica*. In: Pharmacognosy – Medicinal Plants [Working Title]. IntechOpen (2021). https://www.intechopen.com/online-first/a-review-on-the-ethnobotanical-uses-phytochemistry-and-pharmacology-effect-of-luffa-cylindrica

[15] Singh AS, Vellapandian C. Phytochemical studies, antioxidant potential, and identification of bioactive compounds using GC-MS of the ethanolic extract of *Luffa cylindrica* (L.) fruit. Appl. Biochem. Biotechnol. 194(9), 4018–4032 (2022). 35583705 10.1007/s12010-022-03961-1

[16] Bitencourt-Ferreira G, Pintro VO, de Azevedo WF. Docking with AutoDock4. Methods Mol. Biol. 2053, 125–148 (2019). 31452103 10.1007/978-1-4939-9752-7_9

[17] Singh SA, Vellapandian C. The promising guide to LC–MS analysis and cholinesterase activity of *Luffa cylindrica* (L.) fruit using *in vitro* and *in-silico* analyses. Futur. J. Pharm. Sci. 9(1), 33 (2023).

[18] Kumar BS, Anuragh S, Kammala AK, Ilango K. Computer aided drug design approach to screen phytoconstituents of *Adhatoda vasica* as potential inhibitors of SARS-CoV-2 Main Protease enzyme. Life 12(2), 315–339 (2022). 35207602 10.3390/life12020315 PMC8877960

[19] Begum RF, Mohan S. Insights into vitamin E with combined oral contraceptive on *INSR* gene in PCOS by Integrating *in silico* and *in vivo* approaches. Appl. Biochem. Biotechnol. doi: 10.1007/s12010-023-04710-8 (2023) (Online ahead of print). 37610513

[20] Pires DEV, Blundell TL, Ascher DB. pkCSM: predicting small-molecule pharmacokinetic and toxicity properties using graph-based signatures. J. Med. Chem. 58(9), 4066–4072 (2015). 25860834 10.1021/acs.jmedchem.5b00104 PMC4434528

[21] Daina A, Michielin O, Zoete V. SwissADME: a free web tool to evaluate pharmacokinetics, drug-likeness and medicinal chemistry friendliness of small molecules. Sci. Rep. 7(1), 42717 (2017). 28256516 10.1038/srep42717 PMC5335600

[22] Moka MK, Singh AS, Sumithra M. Computational investigation of four isoquinoline alkaloids against polycystic ovarian syndrome. J. Biomol. Struct. Dyn. 1–13 (2023). https://www.tandfonline.com/doi/full/10.1080/07391102.2023.2222828 10.1080/07391102.2023.2222828 37315995

[23] Hardjono S, Siswandono S, Andayani R. Evaluation of N-benzoylthiourea derivatives as popssible analgesic agents by predicting their hysicochemical and pharmacokinetic properties, toxicity, and analgesic activity. Indones J. Biotechnol. 22(2), 76 (2018).

[24] Ayati A, Falahati M, Irannejad H, Emami S. Synthesis, *in vitro* antifungal evaluation and *in silico* study of 3-azolyl-4-chromanone phenylhydrazones. DARU J. Pharm. Sci. 20(1), 46 (2012). 10.1186/2008-2231-20-46 PMC3556043 23351328

[25] Filimonov DA, Lagunin AA, Gloriozova TA et al. Prediction of the biological activity spectra of organic compounds using the Pass Online web resource. Chem. Heterocycl. Compd. 50(3), 444–457 (2014).

[26] Filimonov DA, Rudik AV, Dmitriev AV, Poroikov VV. Computer-aided estimation of biological activity profiles of drug-like compounds taking into account their metabolism in human body. Int. J. Mol. Sci. 21(20), 7492 (2020). 33050610 10.3390/ijms21207492 PMC7593915

[27] Lagunin A, Stepanchikova A, Filimonov D, Poroikov V. PASS: prediction of activity spectra for biologically active substances. Bioinformatics 16(8), 747–748 (2000). 11099264 10.1093/bioinformatics/16.8.747

[28] Şenkardeş S, Han Mİ, Kulabaş N et al. Synthesis, molecular docking and evaluation of novel sulfonyl hydrazones as anticancer agents and COX-2 inhibitors. Mol. Divers 24(3), 673–689 (2020). 31302853 10.1007/s11030-019-09974-z

[29] Ajala A, Uzairu A, Shallangwa GA, Abechi SE. QSAR, simulation techniques, and ADMET/pharmacokinetics assessment of a set of compounds that target MAO-B as anti-Alzheimer agent. Futur. J. Pharm. Sci. 9(1), 4 (2023).

[30] Daina A, Michielin O, Zoete V. SwissADME: a free web tool to evaluate pharmacokinetics, drug-likeness and medicinal chemistry friendliness of small molecules. Sci. Rep. 7(1), 42717 (2017). 28256516 10.1038/srep42717 PMC5335600

[31] Husain A, Ahmad A, Khan SA, Asif M, Bhutani R, Al-Abbasi FA. Synthesis, molecular properties, toxicity and biological evaluation of some new substituted imidazolidine derivatives in search of potent anti-inflammatory agents. Saudi Pharm. J. 24(1), 104–114 (2016). 26903774 10.1016/j.jsps.2015.02.008 PMC4720031

[32] Ertl P, Rohde B, Selzer P. Fast calculation of molecular polar surface area as a sum of fragment-based contributions and its application to the prediction of drug transport properties. J. Med. Chem. 43(20), 3714–3717 (2000). 11020286 10.1021/jm000942e

[33] Khan T, Dixit S, Ahmad R et al. Molecular docking, PASS analysis, bioactivity score prediction, synthesis, characterization and biological activity evaluation of a functionalized 2-butanone thiosemicarbazone ligand and its complexes. J. Chem. Biol. 10(3), 91–104 (2017). 28684996 10.1007/s12154-017-0167-y PMC5480261

[34] ul Hassan SS, Abbas SQ, Ali F et al. A comprehensive *in silico* exploration of pharmacological properties, bioactivities, molecular docking, and anticancer potential of *Vieloplain F* from *Xylopia vielana* targeting B-Raf kinase. Molecules 27(3), 917 (2022). 35164181 10.3390/molecules27030917 PMC8839023

[35] Madden JC, Enoch SJ, Paini A, Cronin MTD. A review of *in silico* tools as alternatives to animal testing: principles, resources and applications. Altern. to Lab. Anim. 48(4), 146–172 (2020). 10.1177/0261192920965977 33119417

[36] Makhouri FR, Ghasemi JB. *In silico* studies in drug research against neurodegenerative diseases. Curr. Neuropharmacol. 16(6), 664–725 (2018). 28831921 10.2174/1570159X15666170823095628 PMC6080098

[37] Calabrese V, Cornelius C, Dinkova-Kostova AT, Calabrese EJ, Mattson MP. Cellular stress responses, the hormesis paradigm, and vitagenes: novel targets for therapeutic intervention in neurodegenerative disorders. Antioxid. Redox Signal. 13(11), 1763–1811 (2010). 20446769 10.1089/ars.2009.3074 PMC2966482

[38] Tyagi A, Kamal MA, Poddar NK. Integrated pathways of COX-2 and mTOR: roles in cell sensing and Alzheimer's disease. Front. Neurosci. 14, 693 (2020). 32742252 10.3389/fnins.2020.00693 PMC7364283

[39] Heppner FL, Ransohoff RM, Becher B. Immune attack: the role of inflammation in Alzheimer disease. Nat. Rev. Neurosci. 16(6), 358–372 (2015). 25991443 10.1038/nrn3880

[40] Akiyama H. Inflammation and Alzheimer's disease. Neurobiol. Aging 21(3), 383–421 (2000). 10858586 10.1016/s0197-4580(00)00124-x PMC3887148

[41] Walker KA, Ficek BN, Westbrook R. Understanding the role of systemic inflammation in Alzheimer's disease. ACS Chem. Neurosci. 10(8), 3340–3342 (2019). 31241312 10.1021/acschemneuro.9b00333

[42] Calabrese V, Mancuso C, Calvani M, Rizzarelli E, Butterfield DA, Giuffrida Stella AM. Nitric oxide in the central nervous system: neuroprotection versus neurotoxicity. Nat. Rev. Neurosci. 8(10), 766–775 (2007). 17882254 10.1038/nrn2214

[43] Chew H, Solomon VA, Fonteh AN. Involvement of lipids in Alzheimer's disease pathology and potential therapies. Front. Physiol. 11, 598 (2020). 32581851 10.3389/fphys.2020.00598 PMC7296164

[44] Zhu T-B, Zhang Z, Luo P et al. Lipid metabolism in Alzheimer's disease. Brain Res. Bull. 144, 68–74 (2019). 30472149 10.1016/j.brainresbull.2018.11.012

[45] Hone E, Lim F, Martins IJ. Fat and lipid metabolism and the involvement of Apolipoprotein E in Alzheimer'sdisease. In: Neurodegeneration and Alzheimer's Disease. John Wiley & Sons, Ltd, Chichester, UK, 189–231 (2019).

[46] Eckert GP, Müller WE, Wood GW. Cholesterol-lowering drugs and Alzheimer's disease. Future Lipidol. 2(4), 423–432 (2007).

[47] Chen B, Zhao Y, Li W, Hang J, Yin M, Yu H. Echinocystic acid provides a neuroprotective effect via the PI3K/AKT pathway in intracerebral haemorrhage mice. Ann. Transl. Med. 8(1), 6 (2020). 32055597 10.21037/atm.2019.12.35 PMC6995749

[48] Montine TJ, Neely MD, Quinn JF et al. Lipid peroxidation in aging brain and Alzheimer's disease 1,2 1Guest Editors: Mark A. Smith and George Perry 2This article is part of a series of reviews on “Causes and Consequences of Oxidative Stress in Alzheimer's Disease”. The full list of papers may b. Free Radic. Biol. Med. 33(5), 620–626 (2002). 12208348 10.1016/s0891-5849(02)00807-9

[49] Yu H, Li W, Cao X et al. Echinocystic acid, a natural plant extract, alleviates cerebral ischemia/reperfusion injury via inhibiting the JNK signaling pathway. Eur. J. Pharmacol. 861, 172610 (2019). 31425684 10.1016/j.ejphar.2019.172610

[50] Kuratsune H, Yamaguti K, Takahashi M, Misaki H, Tagawa S, Kitani T. Acylcarnitine deficiency in chronic fatigue syndrome. Clin. Infect. Dis. 18(Suppl. 1), S62–S67 (1994). 8148455 10.1093/clinids/18.supplement_1.s62

[51] Calabrese V, Cornelius C, Dinkova-Kostova AT, Calabrese EJ. Vitagenes, cellular stress response, and acetylcarnitine: relevance to hormesis. Bio Factors 35(2), 146–160 (2009). 10.1002/biof.22 19449442

[52] Jung I-H, Jang S-E, Joh E-H, Chung J, Han MJ, Kim D-H. Lancemaside A isolated from Codonopsis lanceolata and its metabolite echinocystic acid ameliorate scopolamine-induced memory and learning deficits in mice. Phytomedicine 20(1), 84–88 (2012). 23079229 10.1016/j.phymed.2012.09.005

